# Macroanatomical, Histological and Microtomographic Study of the Teeth of the Komodo Dragon (*Varanus komodoensis*)—Adaptation to Hunting

**DOI:** 10.3390/biology12020247

**Published:** 2023-02-03

**Authors:** Maciej Janeczek, Karolina Goździewska-Harłajczuk, Ludwika Hrabska, Joanna Klećkowska-Nawrot, Piotr Kuropka, Maciej Dobrzyński, Oleksii Melnyk, Anna Nikodem

**Affiliations:** 1 Department of Biostructure and Animal Physiology, Division of Animal Anatomy, Faculty of Veterinary Medicine, Wrocław University of Environmental and Life Sciences, Kozuchowska 1, 51-631 Wrocław, Poland; 2 Faculty of Veterinary Medicine, University of Environmental and Life Sciences, Kozuchowska 1, 51-631 Wrocław, Poland; 3 Department of Biostructure and Animal Physiology, Division of Histology and Embryology, Faculty of Veterinary Medicine, Wrocław University of Environmental and Life Sciences, Norwida 25, 50-635 Wrocław, Poland; 4 Department of Pediatric Dentistry and Preclinical Dentistry, Wroclaw Medical University, Krakowska 26, 50-425 Wrocław, Poland; 5 Department of Animal Anatomy, Histology and Pathomorphology, National University of Life and Environmental Sciences of Ukraine, Potekhin 16, 03041 Kyiv, Ukraine; 6 Department of Mechanics, Materials and Biomedical Engineering, Faculty of Mechanical Engineering, Wrocław University of Science and Technology, Wybrzeże Wyspiańskiego 27, 50-370 Wrocław, Poland

**Keywords:** anatomy, microstructure, histology, teeth, *Varanus komodoensis*, habitat, hunting

## Abstract

**Simple Summary:**

The goal of this study was a detailed analysis of the mandibular teeth structure of the Komodo dragon (*Varanus komodoensis),* based on macroscopic, histological and computed microtomography examinations. The samples were collected post mortem from two adult female Komodo dragons from Wroclaw Zoo. Macroscopically, each mandibular tooth is laterally flattened and its dental crown has an arcuate shape with the typical denticles of a tooth’s caudal margin. The base of the tooth is filled with abundant plicidentine with secondary lamellae. The cavity in the central part of the tooth is well visible, while collagen fibers fill the spaces between the inner lamellae. In the epiphyseal part, the tooth wall is thin and contains numerous dentin trabeculae that grow into the tooth cavity. The dentine is most developed in the mid-tooth, where numerous tubules are observed. The presence of plicidentine, a small number of odontoblasts and a relatively large amount of adipose tissue cells are typical of the mandibular teeth of *V. komodoensis.*

**Abstract:**

The present study aimed to characterize the macrostructure and microstructure of the mandibular teeth of the Komodo dragon (*Varanus komodoensis*) and the methods it uses to obtain food. Examinations were performed using a stereoscopic microscope, autofluorescence method, histological method and computed microtomography. A detailed macro- and micro-structural description of *V. komodoensis* mandibular teeth were made. The mandibular teeth are laterally flattened along their entire length and the dental crown is hooked caudally. The part of the nasal margin of the tooth crown is irregular, while the caudal margin of the tooth is characteristically serrated, except for the tooth base area. There are longitudinal grooves on the lingual and vestibular surfaces up to the lower third of the tooth height. The mandibular tooth is surrounded by a cuff made of the oral mucosa, containing the opening of the venom gland. In the histological structure of the tooth, the enamel covering the tooth crown and the dentin under the enamel are distinguished. The inside of the tooth, except its basal part, is filled with the tooth chamber, while the inside of the lower part of the tooth is filled with plicidentine, which corresponds to external furrows on the enamel. The plicidentine arrangement resembles a honeycomb. A small amount of dentine folds reach up to the tooth apex. Characteristic features of the structure of the mandibular teeth in *V. komodoensis* may indicate their significant role, in addition to the venom glands, in obtaining food in the natural environment of this species.

## 1. Introduction

Teeth are crucial for food acquisition and processing. They have different shapes and sizes, which is related to the method of feeding, the type of food and behavior [1]. It is assumed that heterodonty (incisors, canines, premolars and molars) is a feature typical of mammals [2]. Extinct heterodontosaurids may be an exception here [3]. The different types of teeth are used to perform different specific functions, and are adapted as such. It is generally accepted that modern reptiles have a homodont tooth type.

This classic division is currently being questioned, as some researchers point to the occurrence of heterodonts also in other taxa [1,4,5,6].

The Komodo dragon, alternatively known as a Komodo monitor, is the largest living lizard today. It is also the apex predator on the islands it inhabits [7,8,9]. It was first described by P. A. Ouwens in 1912. The adult Komodo dragon hunts large prey, such as deer, wild pigs and goats, by ambush. It also attacks domestic animals if it has the opportunity to do so.

It attacks when the victim approaches a distance of about one meter [10]. The Komodo dragon’s teeth damage the integument of the victim’s body, which not only causes damage in the form of damage to the skin, hypodermis, muscles, blood vessels and nerves, but also penetrates tissues with the venom produced by the venom glands located near the mandible [11]. The bite force is an important value for a predator, but the Komodo dragon’s bite force is relatively low, and while feeding, it avoids the contact of the teeth with the skeletal elements. Its teeth are adapted to cut soft tissues, thus the microstructure of the teeth cause additional injury [12]. It can, therefore, be concluded that the hunting strategy is much more important than the bite force alone. It seems that the main role in killing the Komodo dragon’s prey is played by venom. The composition of venom can act the same as, for example, in *Varanus varius* [11]. The previous theory about the leading role of bacteria inhabiting the Komodo dragon’s mouth and contributing to the death of the victim seems to be out of date [10,13,14]. Of course, it cannot be ruled out that the oral bacteria of the predator also contribute to the death of the victim, causing infection and potentially even sepsis [15].

Due to scarce data in the literature on the characteristics of the Komodo dragon’s teeth microstructure, the primary aim of this study is conducting the macroscopic, computed microtomographic and histological examinations of the teeth of this species in the context of their methods of obtaining food. Because of the presence of the venom glands in the *V. komodoensis,* we wanted to check whether there are any structures for its escape. We also address the question of whether there are some specific features of the *V. komodoensis* teeth microstructure that support its hunting strategy?

## 2. Materials and Methods

### 2.1. Sample Collection for Macroscopic and Stereoscopic Examinations

The mandibular teeth of two adult female Komodo dragons (*Varanus komodoensis*), obtained from the Wrocław Zoological Garden in Poland, were used in the study. Both females were 7 years old. According to the Red List of Threatened Species (IUCN), the Komodo Dragon is currently an endangered species [16]. The material for the study was collected post-mortem. The cause of death of the animals was not related to pathological changes in the oral cavity. Half of the mandible was collected for the exemplary X-ray of the mandibular teeth and their connection with the bone. Then, the mandibular teeth were collected in consecutive order: six teeth (3 teeth from each animal), for macroscopic and stereoscopic examinations. From these previously collected materials, the following analyses were performed: one tooth from each animal was used for autofluorescence and histological examinations (2 teeth), and one tooth from each animal was evaluated for computed microtomography (2 teeth). We did not analyze maxillary teeth in this study, which will be discussed in the future work. 

The macroscopic study was conducted with a stereoscopic Zeiss Stemi 2000-C microscope (Carl Zeiss, Jena, Germany).

### 2.2. Autofluorescence Analysis

After fixation in a 4% buffered formalin solution, the material was rinsed in running water, dehydrated in an acetone series and dried. Autofluorescence analysis was performed using a Nikon 80iEclipse microscope (Nikon Instruments, Melville, NY, USA) (UV-2A filter, EX 330-380, DM 400, BA 420).

### 2.3. Histological Analysis

After collection, the material was fixed in a solution of 4% buffered formalin and then routinely decalcified in a mixture of formic acid and sodium citrate. The material was then rinsed in running water for 24 h, dehydrated in an alcohol series and embedded in paraffin. Sections 8-μm-thick were cut transversely to the long axis of the tooth. Histological analysis was performed using a Nikon 80iEclipse microscope (Nikon Instruments, Melville, NY, USA) with transmitted polarized light.

### 2.4. Computed Microtomography

Two teeth were examined during the computed microtomography examination. Measurements of the density and geometrical parameters of the teeth were carried out with the use of CTAn and DataViewer programs on reconstructions, obtained with the use of the 1172 SkyScan, Bruker^®^ (Kontich, Belgium) computer microtomography. The samples were recorded with a resolution of 3 µm, with the following lamp parameters: 100 kV/100 µA, and a full 360° rotation (an exposure time of 690 ms; with extra Al filter). The analysis occurred in the Department of Mechanics, Materials and Biomedical Engineering, Faculty of Mechanical Engineering, Wroclaw University of Science and Technology.

## 3. Results

### 3.1. Macroscopic Analysis and X-ray Analysis

**Figure 1:**
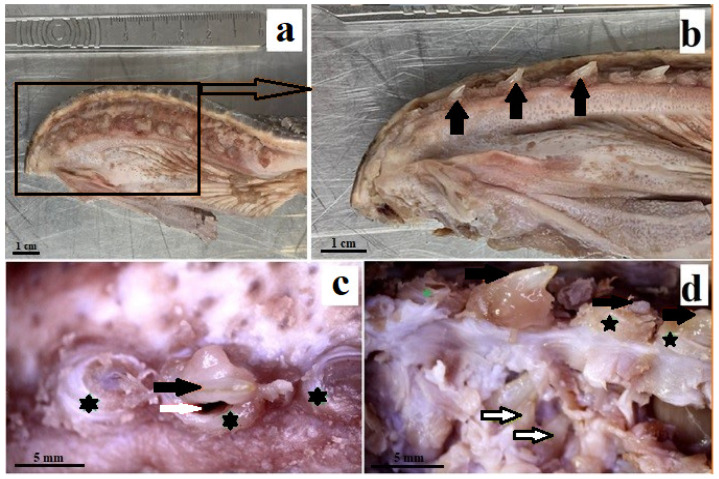
Photomacrographs of the mandibular teeth of the Komodo dragon (*Varanus komodoensis*). (**a**)—location of teeth in the mandible; (**b**)—mandibular teeth (black arrows), teeth bent caudally; (**c**)—enlargement of the mandible with three cuffs around the teeth, the centrally located tooth is surrounded by a cuff (black asterisk) with a visible groove around the tooth (white arrow); (**d**)—enlargement of the mandibular region, where several are visible teeth, the larger one surrounded by a cuff (black arrows), and the smaller one located lower (black and white arrows), visible after removal of the mucous layer.

**Figure 2:**
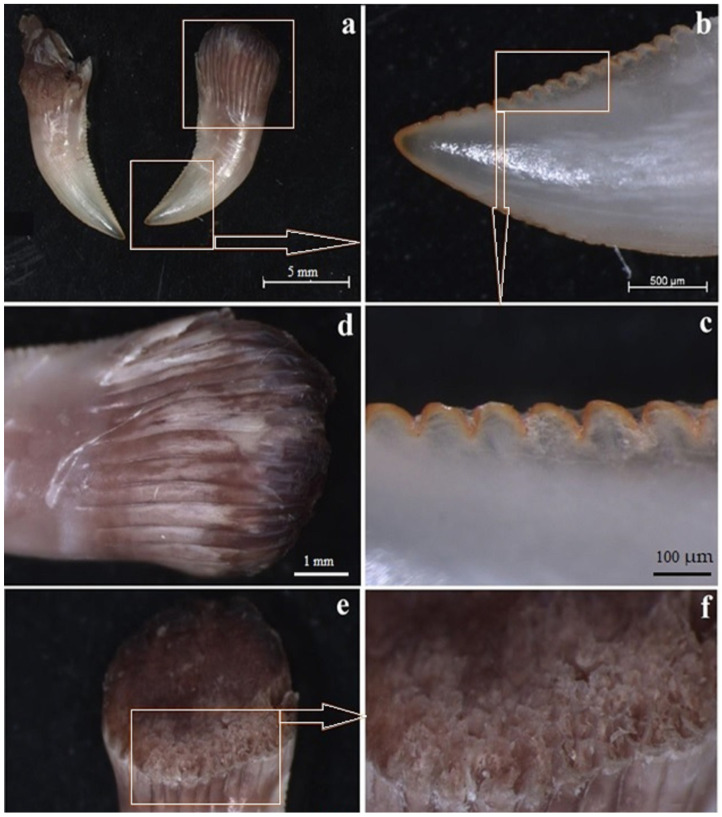
Macroscopic analysis of the mandibular teeth of the Komodo dragon (*Varanus komodoensis*). (**a**)—teeth laterally flattened, tooth crown hooked caudally; (**b**)—serrated distal edge of the tooth; (**c**)—magnification of the distal serrated edge of the tooth; (**d**)—vertically oriented grooves in the enamel protruding into the tooth; (**e**)—densely packed plicidentine; (**f**)—plicidentine enlargement with secondary lamellae merging with adjacent lamellae.

**Figure 3:**
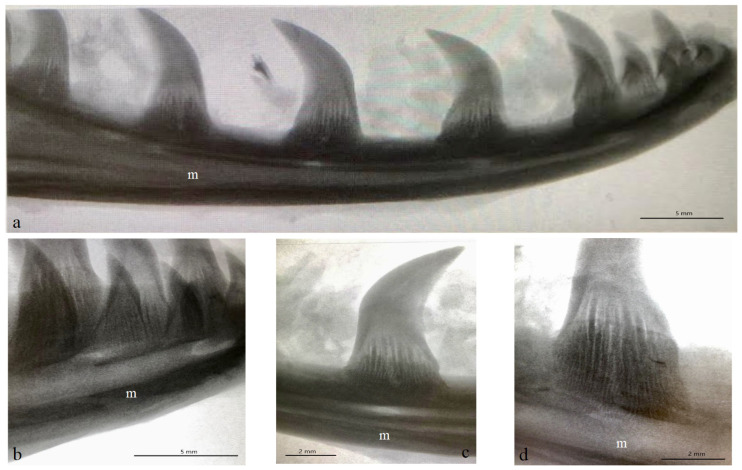
Exemplary X-rays of the mandible of the Komodo dragon (*Varanus komodoensis*) with the mandibular teeth. (**a**)—lateral view of the mandible (right side) with the caudally hooked mandibular teeth, each tooth with vestibular surface, as well as mesial and distal margins, scale bar: 5 mm; (**b**)—magnification of the lateral view of the mandible and mandibular teeth, scale bar: 5 mm; (**c**)—magnification of the mandibular tooth (the mesial and distal margins) and its connection with the mandible, scale bar: 2 mm; (**d**)—magnification of the mandibular tooth without the apical part of the tooth (with well-visible vertically oriented grooves in the enamel protruding into the tooth), scale bar: 2 mm. m—mandible.

The Komodo dragon’s teeth are laterally flattened along their entire length (Figure 1 and Figure 2a). The dental crown is hooked caudally (Figure 2a and Figure 3). Their caudal margin is serrated (Figure 2a–c). These denticles extend along the entire length of the tooth crown and stop at the tooth base area (Figure 2a–d). The teeth involve the tooth enamel layer. The part of the mesial margin of the tooth crown is not smooth but irregular (Figure 2a,b). At the same time, it does not have such regular teething as on the distal margin. There are longitudinal grooves on the lingual and vestibular surfaces up to the lower third of the tooth height (Figure 2a,d). The lingual and vestibular surfaces of the tooth crown covered with enamel are smooth. In the lower part of the tooth, there are vertically oriented grooves in the enamel over its entire surface, which protrude into the tooth (Figure 2a,d). Each mandibular tooth is surrounded by a cuff made of the oral mucosa, containing the opening of the venom gland (Figure 1). After pressing the venom gland with tweezers, the cuff around the tooth is filled with fluid (Figure 1c). The base of the tooth is filled with plicidentine. Some of this plicidentine produces secondary lamellae that fuses with adjacent lamellae to produce a spatial structure that resembles honeycomb (Figure 2e,f). Some plicidentine reaches the apex of the tooth, but not as much as in the base of the tooth, where it is extremely densely packed (Figure 2e,f), creasing the tooth wall outwards. 

### 3.2. Autofluorescence

**Figure 4:**
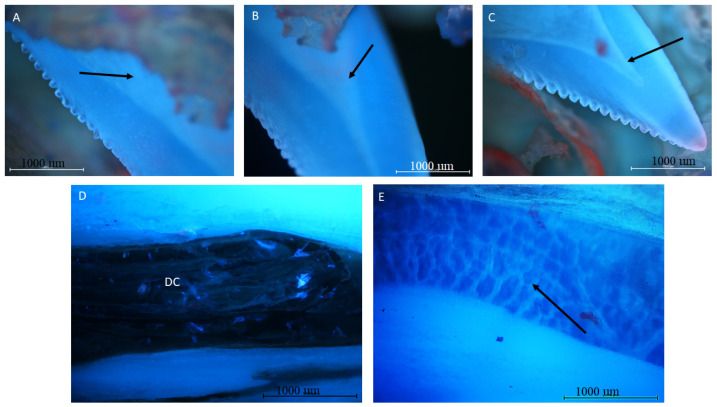
Mandible tooth of a Komodo dragon (*Varanus komodoensis*). (**A**–**C**)—tooth cavity visible in the central part of the tooth (black arrow); (**D**)—root cavity (DC). Visible collagen fibers fill the spaces between the inner lamellae (**E**)—chamber of the middle part of the tooth. Interior filled with adipocytes (arrow). Adipocytes are bigger than the other cells, and contain a single large lipid droplet surrounded by a layer of cytoplasm, and are known as unilocular. The nucleus is flattened and located AT the periphery of the cells. Mag 40× autofluorescence.

The autofluorescence examination revealed the homogenous distribution of mineralized collagen in the tooth wall. No other elements, such unmineralized connective tissue (staining red), are noted. The wall, and level of mineralization, seem to be much weaker than in mammalian teeth. The wall, during the autofluorescence examination, reveals the presence of the empty cavity in the central and apical part of the tooth (Figure 4A–C). On the cross-section of the tooth, numerous collagen fibers fill the spaces between the inner lamellae in the basal part of the tooth, whereas in the middle part, adipocytes are visible. (Figure 4D,E).

### 3.3. Histological Analysis

**Figure 5:**
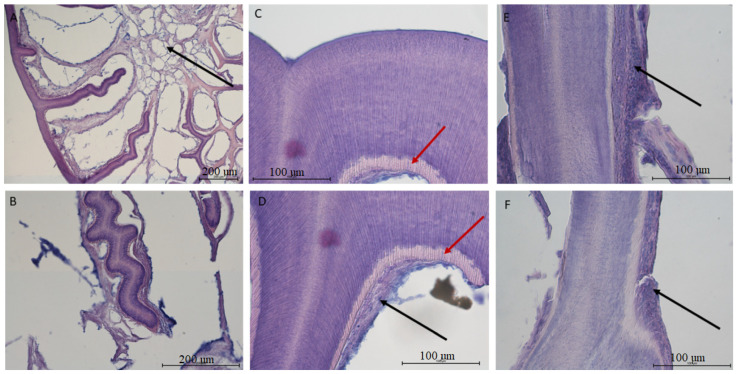
Histological image of the mandible tooth of a Komodo dragon (*Varanus komodoensis*). (**A**,**B**)—the root of the tooth. Visible dentin plates grow into the tooth cavity covered by odontoblasts and the accompanying tooth pulp. In the central part, there are fat cells (black arrow)—Mag 40 and 100×; (**C**,**D**)—tooth wall in the section between the root and the top of the tooth. Visible dentin tubules are arranged in the direction of their synthesis. The dentin is covered with a thin layer of odontoblasts resembling squamous epithelium, covered with collagen fibers, between which there are small blood vessels (black arrow). An area of unmineralized dentin is also visible (red arrow)—Mag 400×; (**E**,**F**)—the image of the tooth wall from the apex. No visible dentinal tubules. Tissues covering the tooth are less reduced (black arrow) in contrast to the wall—Mag 400×.

The tooth of *V. komodoensis* shows great heterogeneity in its structure. In the epiphyseal part, the tooth wall is relatively thin and produces numerous dentin trabeculae, growing into the tooth cavity (Figure 5A). These trabeculae are covered with odontoblasts, taking the shape of a cubic or flat epithelium. Just above them lie collagen fibers accompanied by blood vessels (Figure 5). The inside of the tooth cavity in this area is filled with fat cells. In the intermediate and apical portions, the connective tissue in the central areas completely disappears and remains as a layer covering the dentine, while the interior becomes hollow (Figure 5). Interestingly, the dentine seems to be most developed in the mid-tooth, where there are numerous tubules in the dentine; these are remnants of odontoblast processes. There is also a characteristic line indicating the area of unmineralized dentin (Figure 5C,D). The features described above become weak or completely invisible in the apical part. The very structure of the saw-like ridges on the surface of the tooth is the result of the tooth wall corrugating outwards.

### 3.4. Computed Microtomography

**Figure 6:**
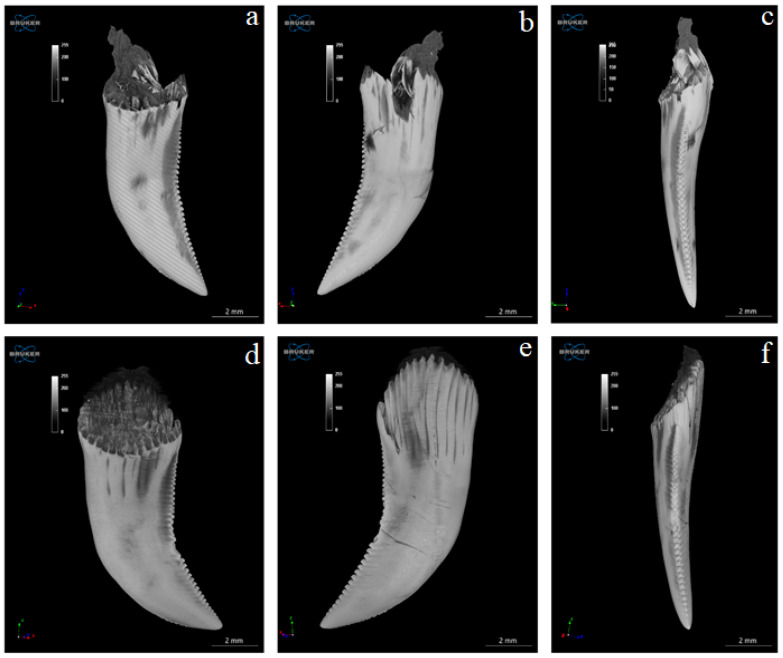
Microtomography analysis of Komodo dragon (*Varanus komodoensis*) 2 teeth (**a**–**f**) in a different orientation. (**a**,**b**)—both surfaces (vestibular and lingual) of the same tooth and (**c**)—its distal edge. (**d**,**e**)—both surface (vestibular and lingual) of the second tooth and (**f**)—its distal edge. Shallow grooves visible in the apical part of the tooth on its mesial margin and deeper, numerous grooves present on the distal margin of the tooth. Note the carina of the distal margin of the tooth (**a**–**f**).

**Figure 7:**
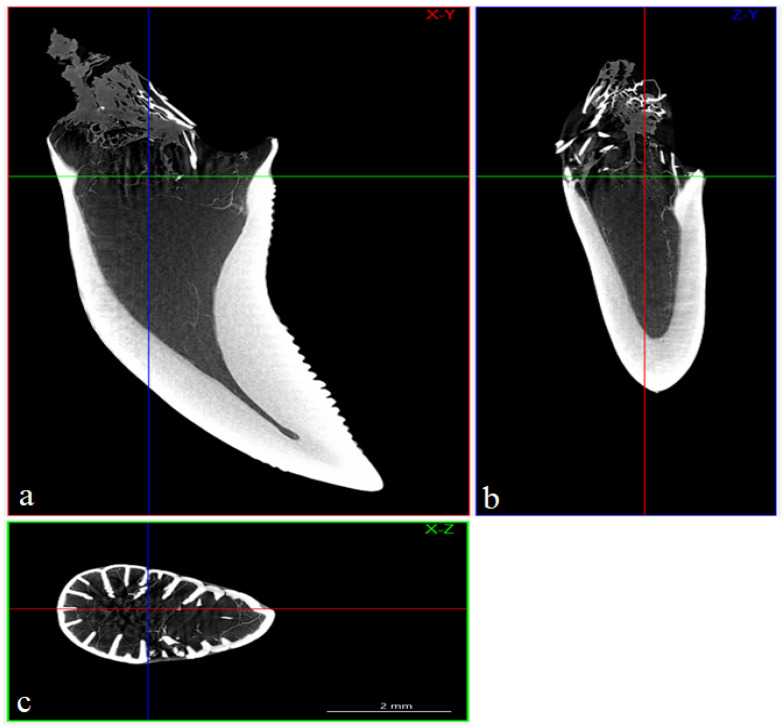
Microtomography analysis of the mandibular teeth of the Komodo dragon (*Varanus komodoensis*). Sample sections (**a**)—coronal (X-Y) (red color), (**b**)—sagittal (Z-Y) (blue color) and (**c**)—transaxial (X-Z) (green color) of the tooth with well-defined tooth cavity. Scale bar: 2 mm.

**Figure 8:**
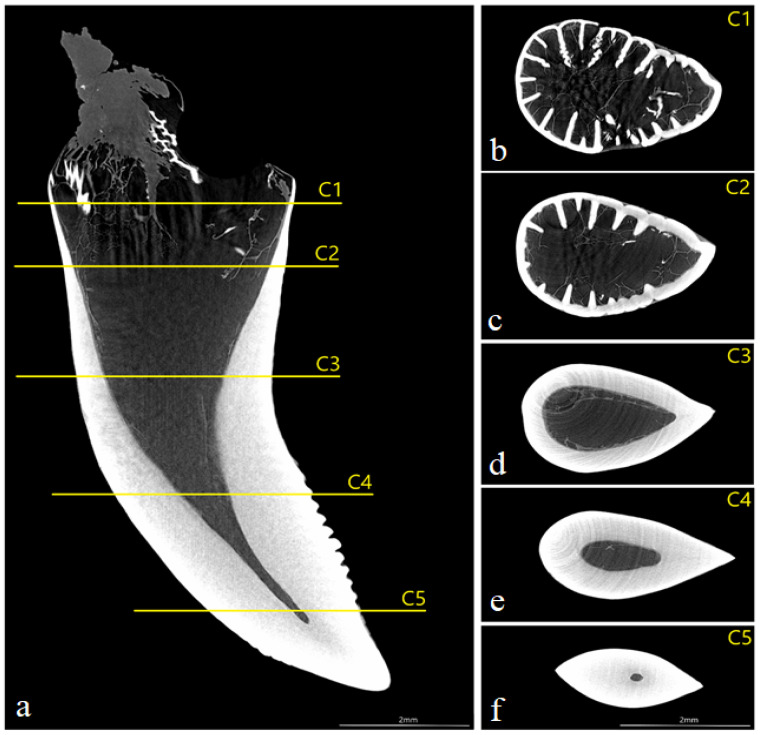
(**a**)—coronal section of mandibular tooth; (**b**)—dentine ridges with a branched structure (main ridges give off secondary ridges that connect—transaxial section—C1), dentine—white; (**c**)—transaxial section of the toot at level C2, note the presence of branched structure; (**d**)—transaxial section of the tooth at level C3, note the absence of branched structure; (**e**)—transaxial section of the tooth at level C4, note the absence of branched structure; (**f**)—transaxial section of the tooth at level C5 in the most apical part of the tooth, note the presence of a very small tooth cavity. Scale bars: (**a**–**f**): 2 mm.

**Figure 9:**
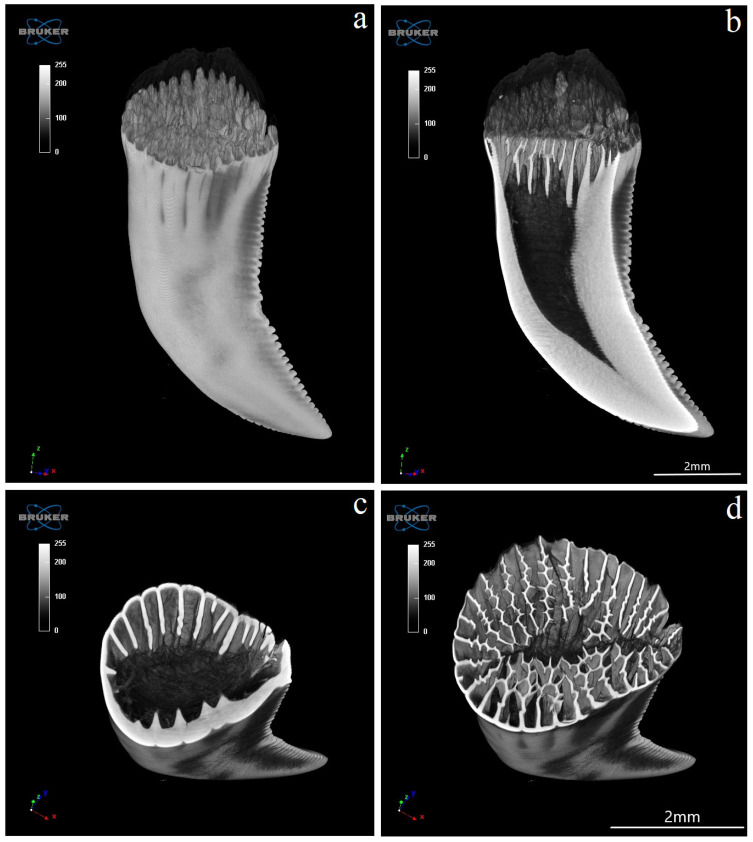
Microtomography analysis of Komodo dragon (*Varanus komodoensis*) mandibular teeth. (**a**)—lateral view with well-visible shallow grooves in the apical part of the tooth on its mesial margin and deeper, numerous grooves present on the distal margin of the tooth; (**b**)—tooth cavity in the transaxial plane, note the presence of a tooth cavity and many serrations of the distal margin; (**c**,**d**)—transaxial section, note plicidentine producing numerous secondary lamellae (**d**) (level C1 from Figure 8) which merge.

**Figure 10:**
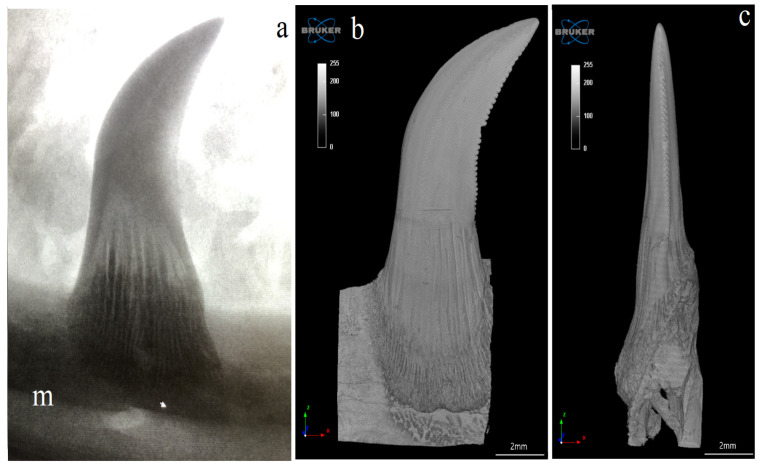
Radiograph and reconstruction of the connection of the mandible bone with the tooth of the Komodo dragon (*Varanus komodoensis*). (**a**)—radiograph of the caudally hooked tooth. m—mandible; (**b**)—vestibular surface of the mandibular tooth with mesial and serrated distal edges, note the connection to the mandible; (**c**)—distal edge of the mandibular tooth with carina.

**Figure 11:**
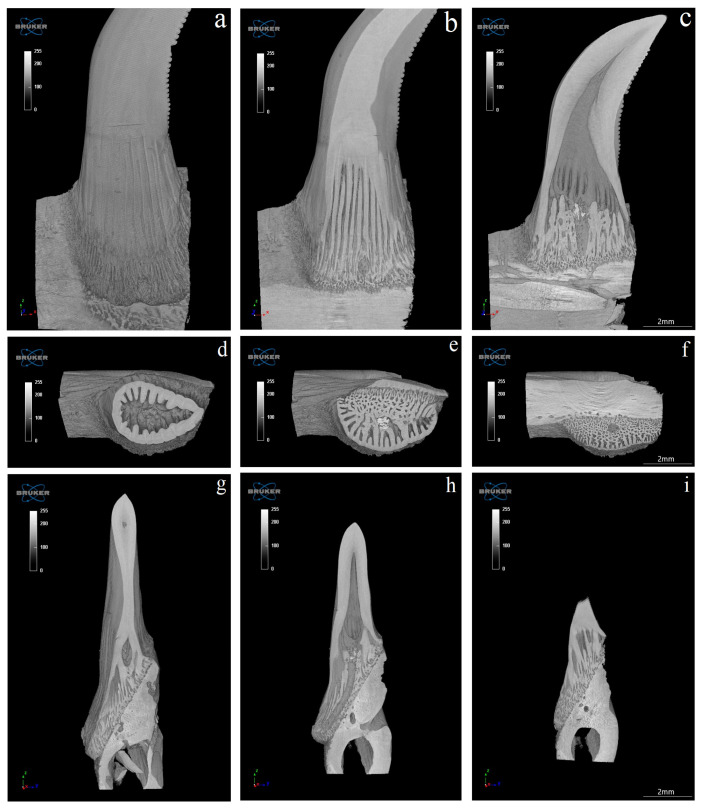
Connection of the mandibula and manidbular tooth of the Komodo dragon (*Varanus komodoensis*)— sections in the coronal (X-Z) (**a**–**c**), transaxial (X-Y) (**d**–**f**), and sagittal (Z-Y) (**g**–**i**) planes. Scale bar: 2 mm.

**Figure 12:**
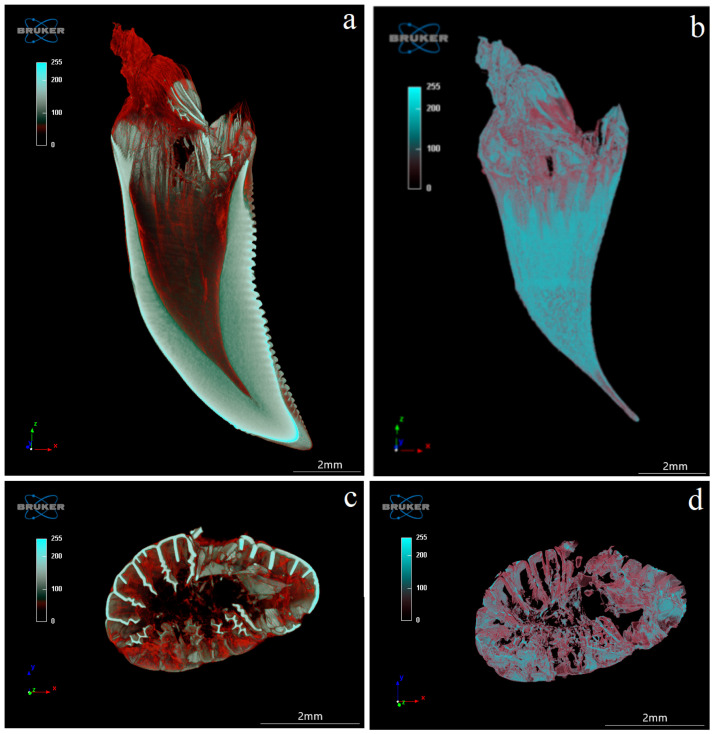
Coronal (X-Z) (**a**,**b**) and transaxial (X-Y) (**c**,**d**) sections of the mandibular tooth of the Komodo dragon (*Varanus komodoensis*). (**a**)—connective tissue and blood vessels stained in red, adjacent to the dentine (white); (**b**)—tooth cavity with connective tissue and blood vessels stained in red; (**c**)—note plicidentine producing numerous secondary lamellae, and blood vessels stained in red; (**d**)—connective tissue with blood vessels. Scale bar: 2 mm.

**Figure 13:**
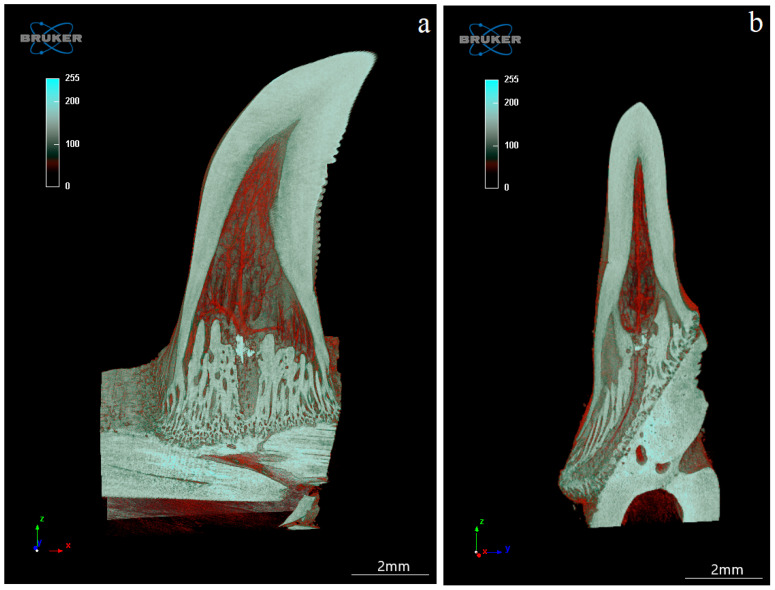
Coronal section (X-Y) (**a**) and sagittal section (Z-Y) (**b**) of the mandibular tooth, together with connective tissue and blood vessels stained in red. Note the connection of the mandible bone to the mandible tooth of the Komodo dragon (*Varanus komodoensis*). Scale bar: 2 mm.

The study showed that not only the distal margin of the teeth produces a ridged structure, but also that the mesial margin is not smooth. There are also grooves in its apical part, although shallower than those present on the distal margin (Figure 6, Figure 7, Figure 8 and Figure 9). It was found that the long axis of the tooth is not straight, but has the character of an arc; therefore, the serrated distal edge of the tooth is not arranged in a straight axis, but is also arcuate or semicircular (Figure 6, Figure 7, Figure 9a,b, Figure 10 and Figure 11). It was observed that the dentine ridges form a branched structure, and the primary ridges give off secondary ridges that can connect with adjacent dentin ridges (Figure 9). The tooth chamber in the apical part narrows significantly, not reaching the very tip of the tooth, and the layer of hard tissues is thicker than in the middle and basal part of the tooth (Figure 7 and Figure 8). The connective tissue and blood vessels adjacent to the dentine of the tooth are defined (Figure 12 and Figure 13).

## 4. Discussion

The teeth of the Komodo dragon are referred to as ziphodont [5], meaning “sword tooth”; it is characterized by labio-lingually compressed, distally curved, serrated crowns. The serrated distal margins have a true series of individualized denticles [4,5,17]. The term was first introduced by O.C. Marsh when describing an eocene crocodilian [12]. It is known that the majority of carnivorous archosaurs in the Mesozoic era possessed it, including Theropoda, the majority of Crurotarsi, and basal Archosauria [3,18]. This type of tooth has also been described in the Permian pelycosaur *Dimetrodon* [18]. In reptiles occurring today, this type of tooth is extremely rare and is restricted to representatives of the family Varanidae [13]. Teeth of modern crocodiles and canines of prey mammals are robust and conical, and have no true denticles [4]. However, although some species of sharks, i.e., very primitive fish, have denticulate crowns, as do fossil mammals, such as saber-tooth cats; these teeth are not classified as ziphodont due to size and/or shape differences in the dentition [12].

Unlike other reptiles with venom glands, lizard teeth do not have structures that facilitate venom drainage, such as those of helodermatid lizards [11]. It should be noted, however, that each tooth of the lizard mandible is subordinated to the mouth of the duct leading out of the venom gland. This opening is located in the cuff of the mucosa surrounding the tooth. This may be important in facilitating the penetration of the venom into the victim’s body. When the tweezers were applied to the venom gland, the venom was released from the cuff surrounding the tooth. However, it cannot be ruled out that the mechanism of release of the contents of the venom glands is different from the pressure exerted by the victim’s tissues. Certainly, the intensively serrated distal edge of the tooth contributes to the good penetration of the victim’s tissues by the venom, but the irregular mesial edge of the teeth also contributes, tearing the soft tissues. During an attack, the Komodo dragon bites the victim and pulls it backwards [13]. Fry et al., 2009 [15] pointed out the similarity of the killing technique used by the Komodo dragon and some sharks and *Smilodon fatalis*. 

The neck and other postcranial muscles play an important role in the killing and subsequent food intake. Teeth and the serious damage they inflict, as well as postcranial input, play an essential role in the effectiveness of the Komodo dragon [18,19]. The Komodo dragon is the largest living lizard [7]. These animals inhabit five islands in Eastern Indonesia [20]. In their habitat, Komodo dragons are apex predators [8,15,21,22]. The diet of adult monitor lizards includes large animals such as rusa deer, feral pigs and water buffalo [23]. Although the prey of Komodo dragons is large animals, the jaw grip of these reptiles is relatively small [5,15,24]. For example, it is 6.5 times smaller than the jaw grip of *Crocodylus porosus*, which also preys on large animals [15]. Studies have shown that monitor lizards do not crush or gnaw bones and joints, being satisfied with the meat of their victims [12]. It seems that the structure of its teeth does not allow such an action, and attempting to crush would result in a fractured tooth. In the histological structure of the tooth, the enamel covering the tooth crown and the dentin under the enamel can be distinguished. The inside of the tooth, except its base part, is filled with the tooth chamber. The inside of the lower part of the tooth is filled with plicidentine, which corresponds to the outer furrows on the enamel. A small number of dentine folds reach the apex of the tooth. Plicidentines thus limit the reach of the dental pulp from below. This phenomenon occurs in fish, amphibians and some reptiles [25]. In the crocodile, the inside of the tooth base is empty [26,27]. 

The presence of plicidentines in monitor lizards was also confirmed and was considered to be a synapomorphy of Varanidae [25]. Reptile enamel does not contain enamel prisms typical for mammals, which is why it is referred to as “prismless enamel” [28]. The tooth chamber does not contain the dental pulp typical of mammals, but instead plicidentine, a small number of odontoblasts and a relatively large amount of adipose tissue cells. The absence of a dental pulp prevents the onset of dentin-forming processes and thus the formation of secondary dentin, as well as the perception of pain stimuli. It can be said that the repair processes in such a tooth are impossible; however, the damaged tooth can be replaced by another one. A crocodile 4 m long was found to have had its teeth replaced 45 times [27]. A similar process appears to be relevant in Komodo Dragons. The morphology of the Komodo dragon’s teeth is adapted to inflict deep, extensive wounds, and the degree of damage to the victim’s tissues allows the proper penetration of the venom. This allows the venom to begin its destructive action and the predator to follow the weakening prey until it falls. While it may be considered that the teeth of Komodo Dragons are evolutionarily archaic and virtually non-existent outside of Varanidae, they are fully functional, making them excellent predators in the environments they inhabit. 

The mechanism of venom flow from the glands through the surface of the teeth into the tissues of the victim requires further study. It is not known whether the venom is extruded mechanically during the bite or if a reaction occurs, or whether the venom is injected through the action of the gland musculature itself or through its outgrowth segments. It is not explained to what degree the venom assists in incapacitating the victim. Thus, the histological structure of the venom glands and their excretory sections also requires further research.

## 5. Conclusions

According to the macroscopic and microscopic analyses of the mandibular teeth structure of the *V. komodoensis* we concluded the following:

- all the teeth are laterally flattened along their entire length and their crows are hooked caudally;
- the distal margin of each tooth is serrated, apart from the base area;
- the teeth are surrounded by cuffs with visible grooves around the teeth where the oral mucosa is, containing the opening of the venom gland;
- histologically, the teeth of *V. komodoensis* show great heterogeneity in their structure, and plicidentine produces numerous secondary lamellae.

## Data Availability

Not applicable.
